# Isolated Infratentorial Posterior Reversible Encephalopathy Syndrome (PRES) in Nephrotic Syndrome: A Case Report

**DOI:** 10.7759/cureus.55056

**Published:** 2024-02-27

**Authors:** Umair Hamid, Faryal A Umair, Deepak Nair

**Affiliations:** 1 Neurology, OSF Healthcare, Peoria, USA; 2 Neurology, University of Illinois College of Medicine, Peoria, USA; 3 Internal Medicine, Dow University of Health Sciences, Karachi, PAK; 4 Neurology, OSF HealthCare, Illinois Neurological Institute, St. Francis Medical Center, Peoria, USA

**Keywords:** supracerebellar infratentorial approach, infratentorial rpls, reversible posterior cerebral edema syndrome, rpls, posterior reversible encephalopathy syndrome (pres), infratentorial pres

## Abstract

We present a case of infratentorial variant posterior reversible encephalopathy syndrome (PRES), which is a very rare presentation of PRES. Atypical PRES is more common than the typical parieto-occipital PRES. We present a 43-year-old male who presented with acute change in mentation, left gaze deviation, and paraparesis with initial blood pressures of 230/120 with anasarca. In the present admission, his CT showed diffuse infratentorial hypodensity. Computed tomography angiography (CTA) was negative for large vessel occlusion. MRI of the brain without contrast showed fluid-attenuated inversion recovery (FLAIR) change diffusely in the brainstem but also extended to the cerebellum and occipital lobe, along with diffusion restriction seen in different regions, including the brainstem and cortex. The patient improved clinically with the improvement of blood pressure and follow-up imaging in five weeks showed improvement of imaging findings. This presentation helps understand the approach to patients presenting with brainstem edema in the acute phase.

## Introduction

Posterior reversible encephalopathy syndrome (PRES) is a neurological disorder characterized by reversible subcortical vasogenic edema leading to neurological symptoms. Symptoms commonly seen in PRES include seizures, encephalopathy, headache, and visual disturbances. The pathophysiology of PRES is not clearly known, and two opposing theories are commonly cited. The more popular theory suggests severe hypertension exceeding the limits of autoregulation, leading to brain edema. The other theory suggests hypertension leads to cerebral autoregulatory vasoconstriction, ischemia, and subsequent brain edema. Common causes of PRES include renal failure, cytotoxic agents, and autoimmune conditions, and in females, there is a predominance of preeclampsia and eclampsia [1-3].

Atypical PRES is more common than the typical parieto-occipital PRES. However, infratentorial PRES is rare (<10% prevalence) [4]. Atypical areas that PRES can involve include the brainstem, thalamus, basal ganglia, cerebellum, temporal lobe, and frontal lobe in increasing order of involvement, with the brainstem being the most rare involvement. The case presented here represents atypical PRES specifically infratentorial PRES.

## Case presentation

**Figure 1:**
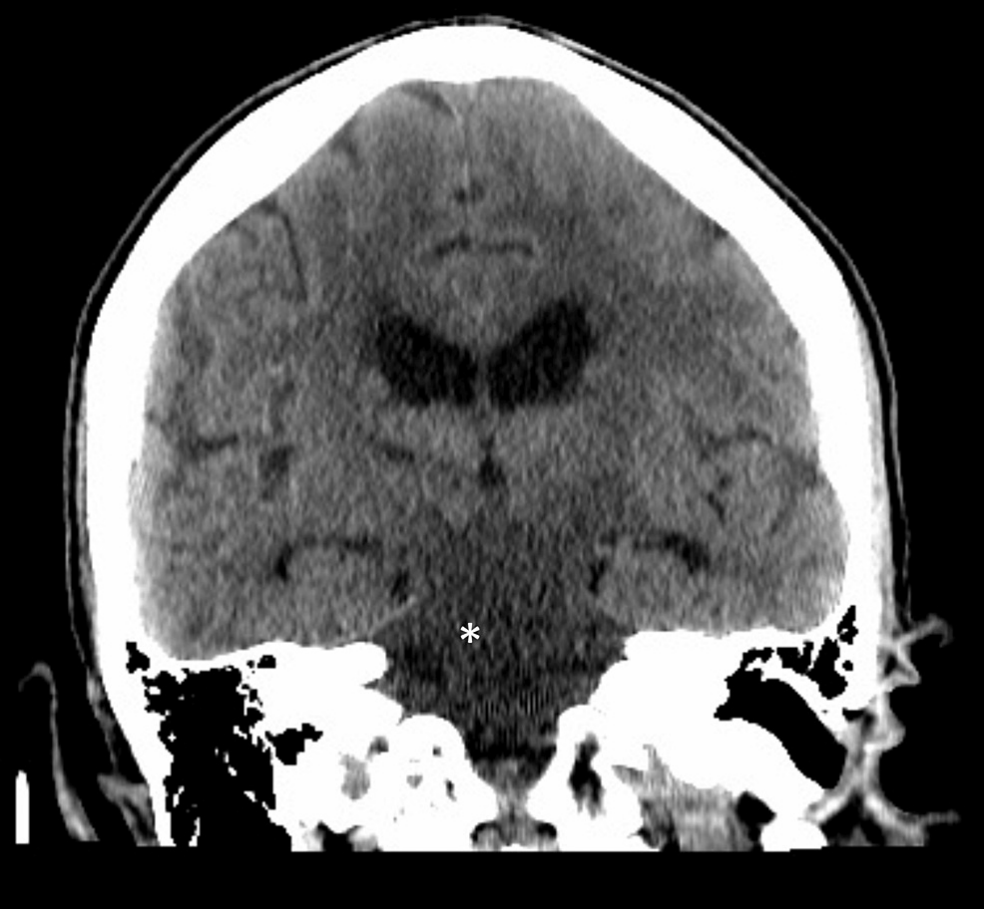
CT head coronal view done four hours after last known normal with hypodensity diffusely involving the midbrain, pons, cerebellar peduncles, and medulla (asterisk).

A 43-year-old man, with a history of uncontrolled hypertension, Klinefelter syndrome with mild intellectual disability, and recently diagnosed nephrotic syndrome (four months prior), was found lying on the ground, with last known normal eight hours ago prior to presentation. His blood pressure on arrival was 210/120 mmHg. He had anasarca on the exam. He was completely disoriented to person, place, and time. Furthermore, his exam showed disorientation to self, place, and time, along with left gaze preference, lower extremity hyperreflexia, leg weakness, and significant truncal ataxia. Lower extremity strength demonstrated 3/5 bilaterally in hip flexion, knee flexion, knee extension, and dorsiflexion. The patient had a non-contrast CT done prior to transfer to our center, which demonstrated diffuse hypodensity in the midbrain, pons, cerebellopontine angle, and medulla (Figure 1).

Based on his presentation, including high blood pressure and the recent diagnosis of nephrotic syndrome, PRES was considered to be differential but did not explain paraparesis (see the Discussion section for further details on localization and differential diagnosis).

**Figure 2:**
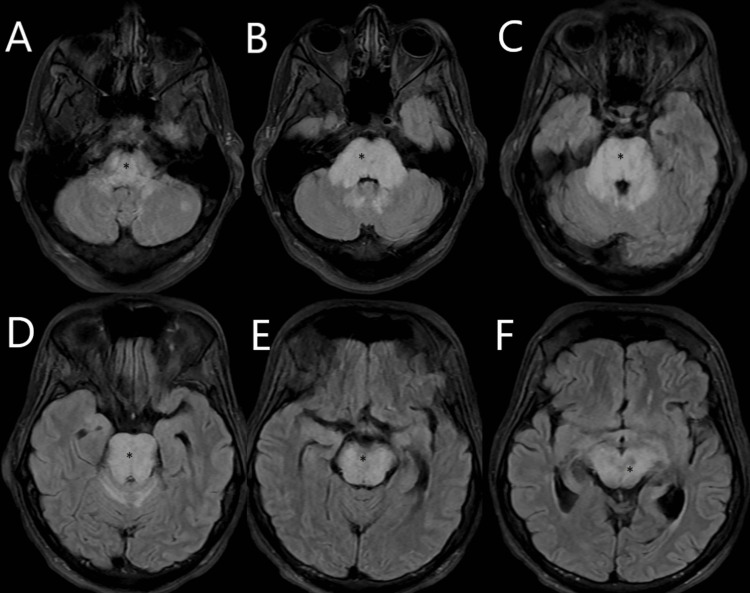
MRI brain (T2-FLAIR) with hyperintensity in the brainstem demonstrating hyperintensity diffusely involving the brainstem structures (asterisk). * hyperintensity representing edema

On arrival, a stat CT angiogram of the head and neck was performed to rule out basilar artery occlusion, which was unremarkable. Stat MRI brain (Figure 2) was performed without contrast in the setting of acute renal failure. MRI demonstrated diffuse T2 hyperintensity in the brainstem and midline cerebellar structures. Diffusion-weighted imaging (DWI)/apparent diffusion coefficient (ADC) maps showed punctate infarcts in the midbrain, pons, and supratentorial including the temporal lobe and thalamus.

A routine EEG was also performed while the patient was still disoriented, and it only demonstrated moderate generalized slowing. Initial lab work demonstrated acute renal injury with creatinine 4.8. His creatinine had been continuously uptrending by 0.5-1.5 points every three months for the past year. Due to renal failure, an MRI with contrast could not be done. Urine analysis also showed 4+ protein and 3+ blood. 

Further testing included CSF analysis, which showed two total nucleated cells, one red blood cell, and protein and glucose were within normal range. Cerebrospinal fluid (CSF) culture, CSF Fram stain, CSF lactate, CSF cytology, Mayo meningitis/encephalitis panel, venereal disease research laboratory (VDRL), HIV, hepatitis B surface antigen (HbsAG), and West Nile antibodies were all unremarkable, making infectious etiology less likely. The autoimmune encephalopathy panel was negative, making paraneoplastic etiology less likely, which was low on differential due to the acuteness of symptomatology. Neuromyelitis optical (NMO) and multiple sclerosis (MS) labs were not obtained due to insufficient samples of CSF. The patient did not present with any lesions of mouth, eyes, or genitals, making Behcet and hence neuro-Behcet less likely.

Autoimmune etiology seemed less likely for two following reasons. The first reason was that the patient improved the next day without any steroids, intravenous immunoglobulin (IVIG), or plasmapheresis, and the second reason is that onset was very quick for any of the differentials to present as autoimmune.

Lab work for vasculitis, including antinuclear (ANA) and antineutrophil cytoplasmic antibodies (ANCA), was negative, making vasculitis less likely. Tox screen was also negative for cocaine, opioids, heroin, and alcohol. The patient did not have any electrolyte abnormalities on presentation or any previous history of one. Assuming that he did not have a history of hypo-/hypernatremia, AIDS, organ transplantation, hemodialysis, or hematological malignancy, osmotic demyelination syndrome (ODS) was ruled out.

**Figure 3:**
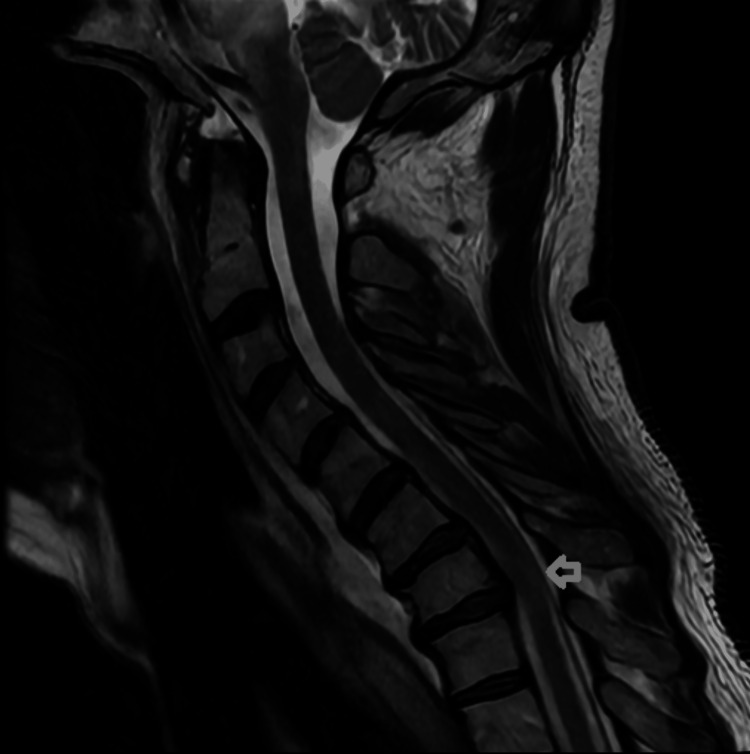
MRI C spine (T2 sequence) sagittal view demonstrating compression of the spinal cord, resulting in increased central and right lateral cord T2 signal due to suspected large right central and subarticular disc protrusion (arrow).

The patient continued to improve over the next one to two days with improvement in his blood pressure. His mentation and gaze palsy were completely resolved. He continued to have some truncal ataxia and paraparesis. In order to work up paraparesis, an MRI C and T spine (Figure 3) was also performed, which demonstrated severe C7 stenosis with T2 spinal cord hyperintensity. This was the confounding factor in his presentation and actually a separate problem unrelated to the main problem.

**Figure 4:**
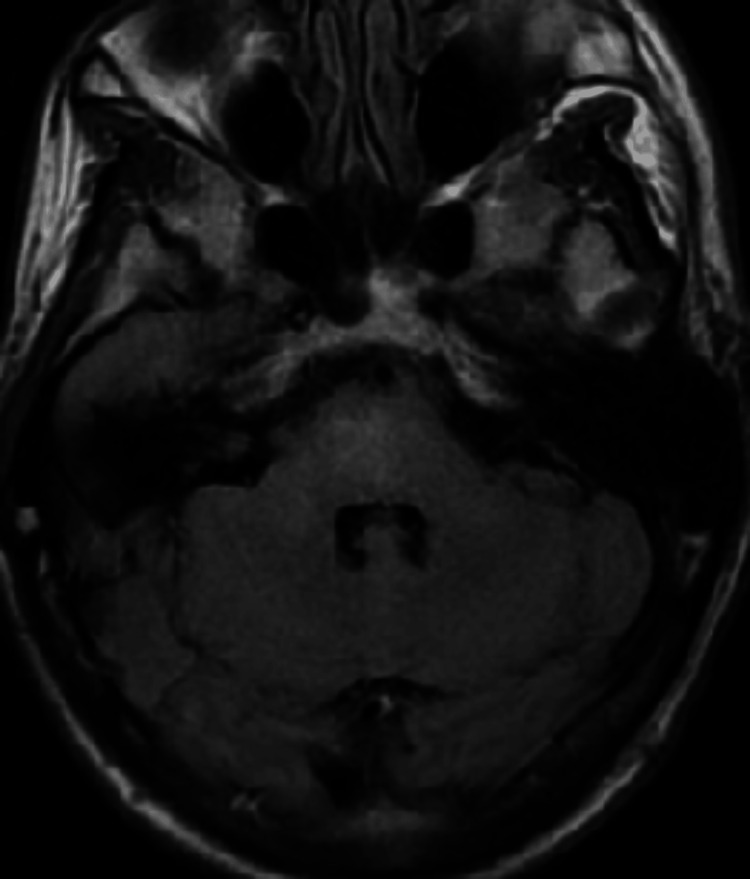
MRI brain axial view of the T2 FLAIR sequence done at week five from the symptom onset.

A repeat MRI brain without contrast was done after five weeks, which demonstrated improvement of T2 hyperintensity in the brainstem (Figure 4). The patient continued to be compliant with anti-hypertensives.

In summary, with very high blood pressure, newly diagnosed nephrotic syndrome, and improvement of his neurological symptoms with good blood pressure control, infratentorial PRES was the working diagnosis.

## Discussion

Localization

The patient in this vignette presents with acute altered mental status, gaze palsy, truncal ataxia, and hyper-reflexic paraparesis. All four problems might not be localizable to one area in the nervous system. Hence, we will need to break it down based on the problem. Gaze palsy is commonly localizable to frontal eye fields (frontal lobes) and paramedian prepontine reticular formation (pons).

Paraparesis can be localized to bilateral corticospinal tracts (frontal lobes, internal capsules, basal ganglia, thalami, brainstem, and spinal cord). Due to hyperreflexia, it is less likely to be localized to the peripheral nervous system. Hence, it is most likely a central nervous system problem. 

Truncal ataxia can be caused by lesions of the cerebellum, especially the midline structures (i.e., vermis, fastigial and interposed nuclei, vestibulocerebellum, and the paravermis). Finally, altered mental status has broad localization supratentorially. A combined localization for acute gaze palsy, truncal ataxia, and paraparesis can be frontal lobes or pons.

Differential diagnosis

Based on presentation, differentials fall in the categories of vascular, infection, trauma, autoimmune, and seizures. From a vascular standpoint, stroke can be a differential, especially on top of the basilar syndrome. Left middle cerebral artery (MCA) syndrome can be considered but would not explain paraparesis. Bilateral anterior cerebral artery (ACA) strokes can cause paraparesis but would not explain gaze involvement.

Trauma can be considered differential based on acute presentation but would affect frontal and temporal lobes, rather than the brainstem, which is not seen on imaging. With disorientation, it is important to know the time of onset, which can be the differentiating factor as seizure becomes a differential in this scenario. Since continuous EEG only demonstrated generalized slowing while the patient had confusion, gaze deviation, and weakness, the seizure was deemed less likely.

Based on imaging, differentials were further narrowed. Other differentials include paraneoplastic encephalitis involving the brainstem, such as anti-Hu (Anti ANNA-1) and anti-Ri (Anti ANNA-2) in breast cancer and anti-NMDA with ovarian teratoma.

Differentials must also include autoimmune encephalitis, such as Bickerstaff encephalitis, chronic lymphocytic inflammation with pontine perivascular enhancement responsive to steroids (CLIPPERS), neurosarcoidosis, neuro-Bechet's, MS, vasculitis, and neuromyelitis optica spectrum disorder (NMOSD). We also include the differential of infectious encephalitis, such as enterovirus, listeria, West Nile virus, and others. Our last mentionable differential would include ODS.

With normal CSF studies, differentials, including autoimmune, viral, and bacterial encephalitis, were ruled out. Additionally, paraneoplastic etiologies and neurosarcoidosis were ruled out based on lab workup. ODS was low on differential due to no recent electrolyte changes, especially with normal sodium levels. CLIPPERS were ruled out since the patient improved without steroids.

With MRI demonstrating significant infratentorial T2 lesions without any enhancement, and the patient spontaneously improving with the resolution of high blood pressures, PRES was the highest on differential.

PRES

PRES is a neurological disorder characterized by reversible subcortical vasogenic edema, leading to neurological symptoms [1]. Symptoms commonly seen in PRES include seizures, encephalopathy, headache, and visual disturbances. The pathophysiology of PRES is not clearly known, and two opposing theories are commonly cited. The more popular theory suggests severe hypertension exceeding the limits of autoregulation, leading to brain edema. The other theory suggests hypertension leads to cerebral autoregulatory vasoconstriction, ischemia, and subsequent brain edema [2]. Common causes of PRES include renal failure, cytotoxic agents, and autoimmune conditions, and in females, there is a predominance of preeclampsia and eclampsia [3].

The case presented here represents atypical PRES specifically infratentorial PRES. Atypical PRES is actually more common than the classic isolated parieto-occipital involvement [4]. However, isolated infratentorial involvement has only been reported a few times in the literature [5]. Less than 10% of all PRES cases can have medullary involvement, and less than 30% can have pons and midbrain involvement [4,6]. DWI is usually negative, but in 15-30% of cases, small foci of restricted diffusion within larger areas of vasogenic edema can be seen. Similarly, PRES-related intracranial hemorrhage is also uncommon. It is seen in 5-15% of all cases. They can be focal parenchymal hematoma, multifocal hemorrhages, and convexity subarachnoid hemorrhage [4].

In this case, there was extensive involvement of the medulla, pons, midbrain, and cerebellar structures with T2 changes. There were no intracranial hemorrhages. The main clinical indicator that favored PRES was spontaneous improvement with the improvement of hypertension. A repeat MRI was performed after five weeks, which showed improvement of the edema. This presentation helps understand the approach to patients presenting with brainstem edema in the acute phase. PRES should be a differential but should not stop clinicians from continuing to do work up for potentially irreversible and debilitating etiologies at the same time.

## Conclusions

This case helps us understand the approach to patients presenting with brainstem edema in the acute phase. PRES should be a differential but should not stop clinicians from continuing to do work up for potentially irreversible and debilitating etiologies at the same time.
